# Outcomes of surgical management for temporomandibular joint ankylosis and pseudoankylosis: a retrospective report of 26 cases

**DOI:** 10.3389/fvets.2025.1616557

**Published:** 2025-06-24

**Authors:** Kristin Kocsis, Stephanie Goldschmidt, Graham Paul Thatcher, Charles Lothamer, Lisa Alexandra Mestrinho

**Affiliations:** ^1^Lone Star Dentistry & Oral Surgery for Animals, Alvin, TX, United States; ^2^Department of Veterinary Surgery and Radiology, University of California, Davis, Davis, CA, United States; ^3^Capital City Specialty & Emergency Animal Hospital, Ottawa, ON, Canada; ^4^Small Animal Clinical Sciences, University of Tennessee College of Veterinary Medicine, Knoxville, TN, United States; ^5^Centre for Interdisciplinary Research in Animal Health (CIISA), Faculty of Veterinary Medicine, University of Lisbon, Lisbon, Portugal; ^6^Laboratório Associado Para Ciência Animal e Veterinária (AL4AnimalS), Lisbon, Portugal

**Keywords:** temporomandibular joint, ankylosis, pseudoankylosis, mandibulectomy, arthroplasty, ostectomy

## Abstract

**Introduction:**

Temporomandibular joint ankylosis and pseudoankylosis are uncommon conditions that can lead to devastating consequences. Surgery is the standard of care with different surgical techniques described.

**Objectives:**

This study compared the outcomes of segmental mandibulectomy (SM), excisional ostectomy (EO), gap arthroplasty (GA), and interpositional arthroplasty (IA) in the surgical management of temporomandibular joint (TMJ) ankylosis and pseudoankylosis in cats and dogs.

**Methods:**

Case accrual was requested from the members of the American Veterinary Dental College listserv. The inclusion criteria included a diagnosis of TMJ ankylosis or pseudoankylosis, confirmed either by helical computed tomography (CT) or cone beam computed tomography (CBCT), surgical treatment, and follow-up information of 2 weeks for short-term complications, 3–6 weeks for medium-term complications, and >4 months for long-term complications.

**Results:**

A total of 26 cases (14 cats and 12 dogs) from 10 institutions were included from 2011 to 2024. Surgical treatment outcomes were categorized with a proposed improvement score classification system based on the percent range of motion (ROM) improvement, requirement for revision surgery, and presence of transiente or permanent complications. Excellent, good, and fair outcomes were observed across all procedure types, with no poor outcomes diagnosed. SM resulted in complications in all cases, with no excellent outcomes. Perioperative complications were rare, with only one case of hypothermia reported in a cat. Conversely, the postoperative complication rate was 50% (13/26) and included neuromuscular issues (19.2%; 5/26), malocclusion (26.9%; 7/26), callus formation not requiring surgical revision (3.8%; 1/26), and re-ankylosis requiring surgical revision (15.4%; 4/26). Surgical revision was only required in patients initially treated with SM and GA.

**Conclusion:**

This study confirms that excellent outcomes are possible for cats and dogs affected by TMJ ankylosis and pseudoankylosis, and that IA may have postoperative advantages compared to GA and SM.

## Introduction

Temporomandibular joint (TMJ) ankylosis is defined as a fibrous or bony fusion of the mandibular head of the condylar process and the mandibular fossa of the squamous part of the temporal bone (1–3). While “true” ankylosis involves the intraarticular structures of the TMJ, pseudoankylosis or “false” ankylosis occurs with fibrous or bony encapsulation of the joint, or structures remote to the joint (4–6). TMJ ankylosis and pseudoankylosis are uncommon conditions that may occur individually or in tandem, and occur secondary to trauma, osteoarthritis, neoplasia, or inflammatory conditions (7–11). These conditions are severely debilitating with potential life-threatening consequences if no intervention is performed, including aspiration pneumonia, impaired heat regulation, or malnutrition. In addition, lack of airway access may negatively impact the patient if an elective or emergency anesthetic procedure is required.

**Figure 1:**
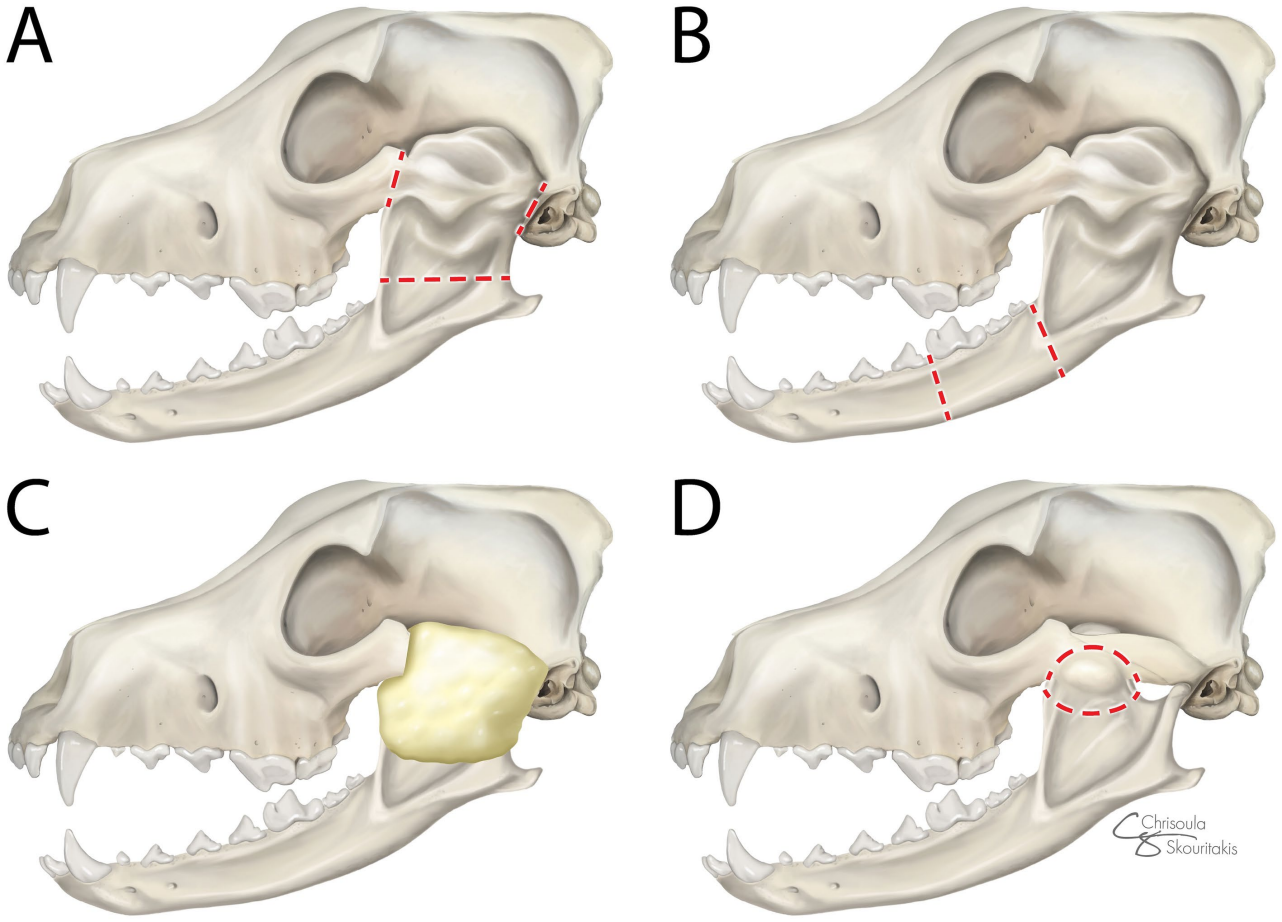
Lateral view of surgical interventions for ankylosis and pseudoankylosis: **(A)** gap arthroplasty (GA)—zygomectomy, coronoidectomy, condylectomy, and removal of the mandibular fossa of the temporal bone, **(B)** segmental mandibulectomy (SM)—excision of a segment of the mandible rostral to the fusion, **(C)** interpositional arthroplasty (IA)—interpositional material application into the surgically created gap following GA, and **(D)** excisional ostectomy (EO)—removal of the bony callus remote to the joint. Dotted red lines denote the margins of the excision.

The standard of care for TMJ ankylosis and pseudoankylosis is surgery to release the fusion and increase the TMJ range of motion. When possible, non-surgical management such as stretching maneuvers and periarticular corticosteroid administration can be attempted, but these results in increased recurrences of TMJ fusion compared to surgery (12). In humans and animals, radical resection, as well as timely intervention, have been shown to play an important role in the prevention of postoperative adhesions and re-ankylosis (13, 14). Gap arthroplasty (GA), which involves zygomectomy, coronoidectomy, condylectomy, and removal of the mandibular fossa of the temporal bone, has been shown to provide favorable results in both humans and animals (14–18) (Figure 1). When interpositional materials such as fat graft and temporalis myofascial graft are placed into the gap, the procedure is referred to as interpositional arthroplasty (IA) (19, 20). Human studies have shown that the application of IA results in increased mouth opening and reduced risk of re-ankylosis when compared to GA (21, 22). In veterinary medicine, both the temporal myofascial flap and fat grafts have been used with anecdotal success, while published data are limited and only includes case reports for this procedure in cats (18, 23). Overall, definitive data are lacking to determine if IA yields better outcomes than GA in veterinary species.

In cases of pseudoankylosis where the TMJ is not directly affected, excisional ostectomy (EO) of the bony callus remote to the joint is a less radical surgical option than GA to restore function. Another alternative surgical treatment for both ankylosis and pseudoankylosis is segmental mandibulectomy (SM), where there is excision of a segment of the mandible rostral to the fusion (Figure 1). This does not improve ROM by addressing restricted motion of the joint, but rather permits mandibular movement from the rostral to the fusion to improve functionality. The primary theoretical benefit of this approach is a decreased risk of intraoperative hemorrhage, while the theoretical negative includes an increased risk of re-ankylosis compared to more radical techniques. Use of SM has been shown to effectively manage unilateral ankylosis in cats (19, 20), but the technique was not compared directly to GA, and clinical outcome data are lacking in dogs, leaving knowledge gaps about its utility. This study aimed to compare the perioperative and postoperative outcomes of SM, EO, GA, and IA for the surgical management of TMJ ankylosis and pseudoankylosis in cats and dogs.

## Materials and methods

**Figure 2:**
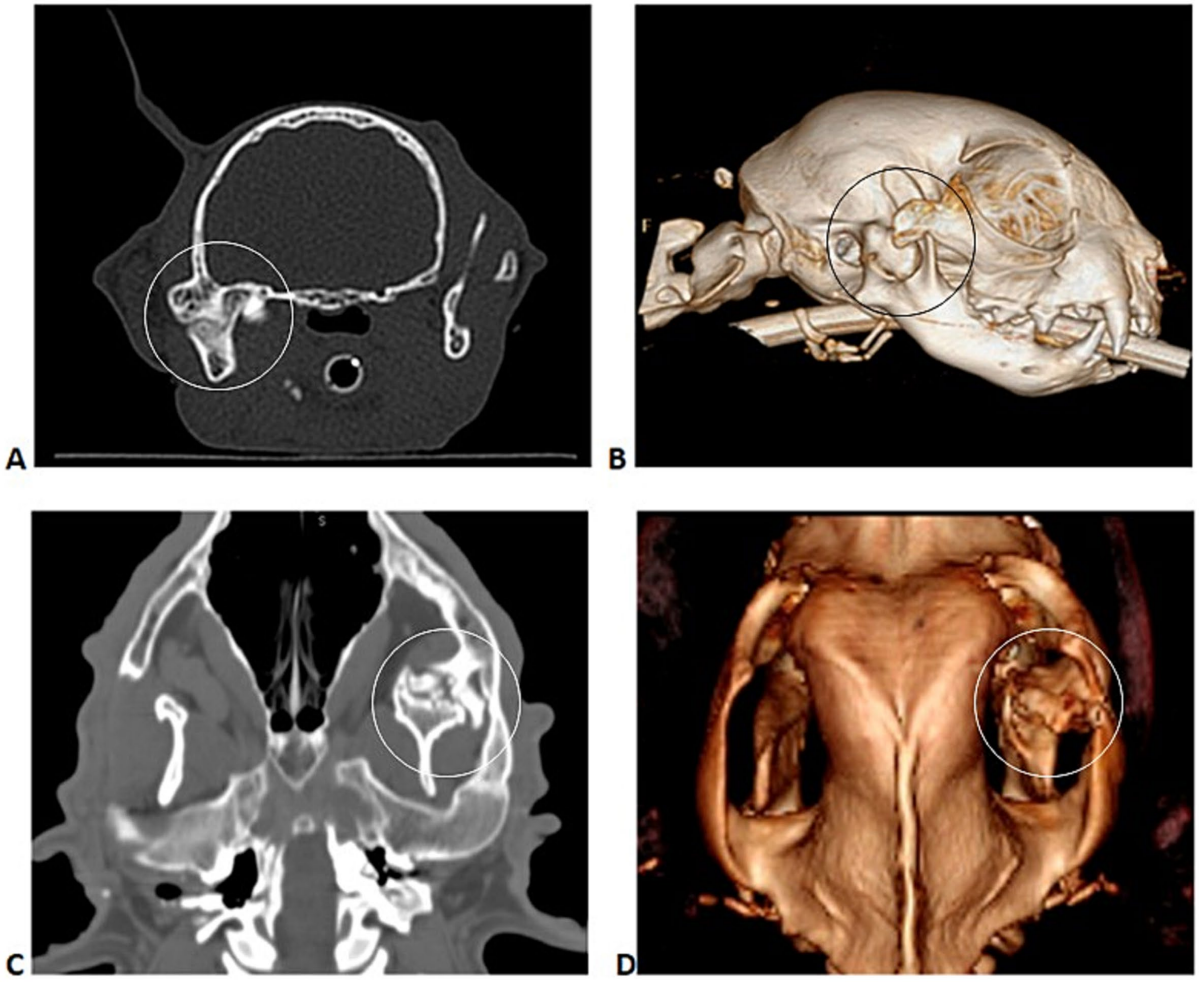
Transverse multiplanar reconstruction (MPR) **(A)** and corresponding 3D volume rendering CT image **(B)** of a cat with right TMJ ankylosis; dorsal CT image **(C)** and corresponding 3D volume rendering CT image **(D)** of a dog with left TMJ pseudoankylosis.

**Figure 3:**
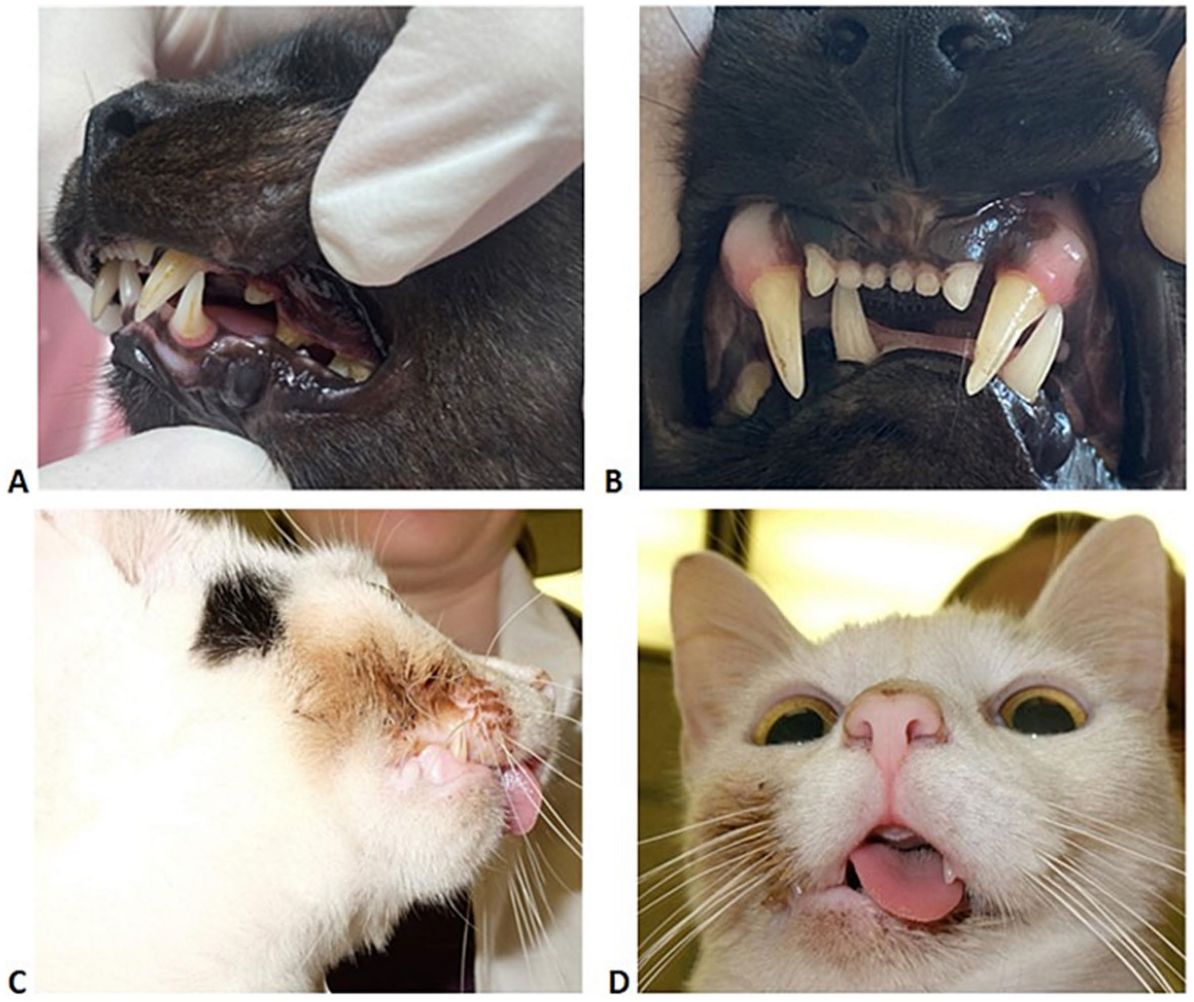
**(A–D)** Represent clinical photographs of post-operative malocclusions following surgical treatment for TMJ fusion in two cats, highlighting the importance of monitoring and surgical intervention for acquired malocclusions. The cats in **A,B** would benefit from either crown height reduction or selective dental extractions to alleviate trauma and discomfort.

Case accrual was solicited from the American Veterinary Dental College members using the members’ listserv. A single-case solicitation email was submitted to the listserve in October of 2023. All received cases were reviewed, and case updates were accepted through February 2025. Inclusion criteria were (1) restricted TMJ range of motion as determined by operator assessment at clinical presentation, (2) confirmation of TMJ ankylosis or pseudoankylosis using helical computed tomography (CT) or cone beam computed tomography (CBCT) (Figure 2), (3) surgical intervention with either SM, EO, GA, or IA, and (4) in person patient outcome assessments performed 2 weeks after surgery or greater. Patient data retrieved included species, breed, gender, and age at presentation. Diagnosis and treatment data included the type of ankylosis diagnosed on CT or CBCT imaging as reported in the patient medical records, skull conformation (mesocephalic, brachycephalic, or dolichocephalic) as determined by the veterinary surgeon, presence of preoperative malocclusions, if surgical planning included 3D and multiplanar reconstruction (MPR) or 3D printing, surgical technique performed, preoperative and postoperative interincisal distance, presence of complications, and if surgical revisions were required. Complications were defined as (1) intraoperative: occurring during surgery, (2) short term: occurring within 2 weeks of postoperative follow-up, (3) medium term: occurring within 3 to 6 weeks of postoperative follow-up, (4) long term: occurring in patients with > 4 months of postoperative follow-up, and (5) transient (resolved) or permanent (did not resolve). Only patients with sufficient in-person follow-up were evaluated for the presence of medium or long-term postoperative complications.

ROM at the initial visit was measured as interincisal distance in millimeters (mm). Postoperative interincisal distance in mm was variably reported, and ROM recovery was determined as an estimated percentage of normal based on recheck evaluation findings by the veterinarian (<20%, 20 to 50%, 50 to 80, and >80%). A postoperative outcome score was developed to include the occurrence of postoperative complications, ROM improvement, and need for revision surgery to better classify the degree of success experienced by canine and feline patients following surgical intervention (Table 1). Score classification required that at least one of the criteria be met.

**Table 1:** Postoperative evaluation criteria for outcome score.

Outcome score	Criteria
Excellent	> 80% recovery of ROM; no short, medium, or long-term transient postoperative complications, no permanent postoperative complications, no postoperative acquired malocclusion
Good	50–80% recovery of ROM, short, medium, or long-term transient postoperative complications (facial nerve damage or pain), minor permanent postoperative complications (local muscle atrophy).
Fair	20–50% recovery of ROM, revision surgery required, resulting in functional improvement, postoperative acquired malocclusion, permanent postoperative complication (facial nerve damage)
Poor	<20% recovery of ROM, revision surgery without functional improvement, permanent complications (pain)

The commercial software Excel (version 16.83 for mac) was used to register and standardize variables and perform graphical representations of results. Statistical analysis was made using a commercial software package (IBM SPSS, version 29 for Mac). The following variables were grouped for the analysis: cases presenting both ankylosis and pseudoankylosis were grouped with cases presenting solely ankylosis, and one case undergoing both IA and EO was included in the group IA. Categorical variables were analyzed using a non-parametric test, chi-square, and Fisher’s exact test. An age interval was defined as ≤ 1 year and 1 year as a categorical variable. A *p*-value of <0.05 was considered significant for a 95% confidence interval.

## Results

A total of 26 cases, including 14 cats and 12 dogs, met the criteria for inclusion. Excluded cases displayed ROM deficits unrelated to ankylosis or pseudoankylosis, or lacked follow-up information. Animals that were surgically treated from the years 2011 to 2024 were contributed by four universities and six privately owned dental surgical centers. The median age of cats was 1 year (range: 4 months to 14 years), with nine cats being 1 year of age or less. Gender distribution was five males and nine females. Cat breeds included 12 Domestic Short Hairs and 2 Persians. The median age of dogs was 1 year (range 4 months to 7 years), and the gender distribution was five males and seven females. Dogs were represented by nine medium to large breeds, one small breed, and two mixed breeds. Preoperative hematology and serum biochemical profiles revealed non-specific abnormalities in all patients. Pseudoankylosis was diagnosed in 11 patients and ankylosis in 15 patients. Diagnosis was associated with species, with cats significantly more likely to have ankylosis than dogs (OR dogs compared to cats: 0.136, 95% CI [0.18, 0.99], *p* = 0.045).

3D printing was performed in addition to 3D and MPR in eight cats and three dogs. The use of 3D printing as a tool for pre-operative planning was not significantly associated with the type of surgery performed (*p* = 0.211), need for revision surgery (*p* = 0.123), ROM improvement (*p* = 0.187), nor the presence of postoperative malocclusion (*p* = 0.100).

Surgical treatments included five SM (three dogs, two cats), seven EO (five dogs, two cats), nine GA (three dogs, six cats), and five IA (one dog, four cats). IA utilized temporal muscle transposition in three cases and local subcutaneous fat in two cases. All IAs were performed in cases of TMJ ankylosis. The surgery technique performed was significantly (*p* = 0.002) associated with the diagnosis; the most common surgery performed in cases of ankylosis was GA, while EO was the most common surgery performed in pseudoankylosis cases (Table 2).

**Table 2:** Complications and patient outcomes observed in a group of 26 dogs and cats treated surgically for temporomandibular joint ankylosis or pseudoankylosis.

	Postoperative complications (*n* = 26)	Postoperative complications short-term follow-up (2 weeks) (*n* = 4)	Postoperative complications medium-term follow-up (3–6 weeks) (*n* = 8)	Postoperative complications long-term follow-up (>4 months) (*n* = 13)	Revision surgery required (*n* = 26)	Patient outcome long-term follow-up (>4 months) (*n* = 13)		
						Fair	Good	Excellent
Procedure performed								
IA	80.0% (4/5)	0.0% (0/0)	100.0% (a) (1/1)	75.0%^a^^,^^d^ (3/4)	0.0% (0/5)	25.0% (1/4)	50.0% (2/4)	25.0% (1/4)
EO	14.30%^a^^,^^d^ (1/7)	0.00% (0/1)	0.00% (0/2)	0.00% (0/3)	0.00% (0/7)	0.00% (0/3)	0.00% (0/3)	100.00% (3/3)
SM	100.0% (5/5)	100.0%^d^ (1/1)	100.0%^b^^,^^c^^,^^d^ (3/3)	100.0%^b^ (1/1)	60.0% (3/5)	100% (1/1)	0.0% (0/1)	0.0% (0/1)
GA	33.30% (3/9)	0.00% (0/2)	50.00%^b^ (1/2)	40.00%^a^^,^^d^ (2/5)	11.10% (1/9)	20.00% (1/5)	20.00% (1/5)	60.00% (3/5)
Diagnosis								
Ankylosis	60.0% (9/15)	20.0% (1/5)	75.0% (6/8)	30.8% (4/13)	20.0% (3/15)	12.5% (1/8)	37.5% (3/8)	50.0% (4/8)
Pseudo-ankylosis	36.4% (4/11)	80.0% (4/5)	25.0% (2/8)	69.2% (9/13)	9.1% (1/11)	25.0% (1/4)	0.0% (0/4)	75.0% (3/4)

a Neuromuscular deficits.

b Re-ankylosis requiring surgical revision.

c Callus formation not requiring surgical revision.

d Acquired malocclusion (including mandibular drift).

Malocclusions were noted preoperatively in 38.5% (10/26; six dogs, four cats) of cases, with 23.1% (6/26) believed by the surgeon to be related to the fusion and included class 4 (*n* = 5) and class 2 (*n* = 1) malocclusions as defined by the AVDC (24). In 11.5% (3/26) of patients, a malocclusion suspected to be unrelated to the fusion was observed (class 3). The suspected origin of one case of class 2 malocclusion was not identified.

Intraoperative and postoperative complications related to the primary surgical intervention were evaluated. Only one intraoperative complication during the initial surgery was reported, which was hypothermia in a 1-year-old cat. Complications encountered during the surgical revision procedures were not uniformly available and were therefore not included in this analysis.

Postoperative complications were seen in 50% (13/26) of all cases. Complications included neuromuscular complications, acquired malocclusions, callus formation not requiring surgical revision, and re-ankylosis with the need for revision surgery (Figure 3). Neuromuscular complications occurred in 19.2% (5/26) of cases. Cases treated with GA, IA, and EO experienced neuromuscular complications, with three experiencing transient facial nerve neuropraxia (one treated with GA, one treated with EO, and one with IA) and two experiencing temporal muscle atrophy reported following IA. The presence of neuromuscular complications was not correlated with diagnosis (*p* = 0.428), but was correlated with the surgery technique (*p* = 0.010). Malocclusions acquired following surgical intervention occurred in 26.9% of cases (7/26), one in IA, three in SM, one in EO, and two in GA. Callus formation not requiring surgical revision was noted in 3.8% (1/26) of cases. This one case was treated with SM. The presence of acquired postoperative malocclusions was not related to diagnosis (*p* = 1.000), the need for revision surgery (*p* = 1.000), ROM improvement (*p* = 0.705), nor surgical technique (*p* = 0.179).

Surgical revision due to re-ankylosis was required in 15.4% of cases (4/26): two cats and two dogs, 75% (3/4) being diagnosed primarily with ankylosis. SM was initially performed in two dogs and one cat, and GA was initially performed in one cat. No cases with IA or EO required revision surgery. The need for revision surgery was significantly associated with the surgical technique (*p* = 0.016), with 60% (3/5) of animals treated with SM requiring surgical revision (Table 2). Surgical revision techniques utilized included two GA, one IA, and one SM. All cases requiring surgical revisions were mesocephalic animals less than 1 year of age. Age interval was not significantly associated with the need for revision surgery (*p* = 0.122). Conversely, surgical technique was significantly associated with the need for a revision surgery (*p* = 0.041). Identification of re-ankylosis and surgical revision occurred within 1 month in three cases, and within 6 months in one case.

Importantly, regarding complications, the follow-up was variable in the study cohort. Specifically, 4 cases had only short-term follow-up (2 weeks), 8 cases had medium-term follow-up (3–6 weeks), and 13 cases had long-term follow-up (> 4 months). To not understate the rate of complications due to lack of follow-up, we have also reported the rate of complications and the type of complications seen in each group based on follow-up time (Table 2). Furthermore, the final outcome score was also only assessed in patients with long-term follow-up (> 4 months, *n* = 13). The outcome was excellent in seven cases, good in three cases, and fair in three cases. Poor outcomes were not registered in this case series.

ROM improvement to varying degrees was noted among all patients. ROM was reported as an interincisal distance in mm in all cases preoperatively. In cats (*n* = 14), the mean initial interincisal opening was 5.71 mm (range <1 to 20 mm). In five cats, postoperative interincisal distance was measured, and the mean improved post-surgical interincisal distance was 33.6 mm (26–40 mm). When postoperative interincisal distance was not measured, ROM was estimated (*n* = 7) or not assessed (*n* = 2). In these cases, veterinarian estimations ranged from 50 to 85% improvement of ROM, normal or near normal ROM, or return to good or normal function, where measurements were not available. In dogs, the mean initial interincisal distance (*n* = 12) was 10.3 (range: 2 to 30 mm). When measured postoperatively (*n* = 8), the mean improved post-surgical interincisal distance was 65.3 (range 50 to 80 mm). In four cases, postoperative interincisal distance was estimated, and ROM improvement ranged from 50% normal to normal.

## Discussion

This study aimed to compare the outcomes of SM, GA, IA, and EO for TMJ fusion (ankylosis and pseudoankylosis) in cats and dogs. In this study, intraoperative complications were rare, being noted in only one case, but postoperative complications were frequent, occurring in 50% (13/26) of all cases, most noted with long-term follow-up. Postoperative complications encountered included 19.2% (5/26) of patients developing neuromuscular compromise, 26.9% (7/26) developing malocclusions, and/or 15.4% (4/26) of patients requiring revision surgery. Regardless of complications, ROM improvement occurred in all patients, and no poor outcomes were registered based on the proposed outcome score.

Contrary to this study, more severe intraoperative complications and poor outcomes were reported in a historical retrospective study where blood transfusion was necessary (25). In the same series, euthanasia was elected in one case due to the severity of the disease, while no such severe endpoints were observed here (25). Although devastating complications appear rare, this may reflect patient selection bias within our current case series, as it is possible that higher-risk patients may not have proceeded with intervention. Additionally, all cases in the previous retrospective analysis came from a university setting, while this case set included a mix of university and private practices. The results of this study support the concept that clients and surgeons should approach this surgery with the expectation that treatment carries a high risk of postoperative complications, but with appropriate patient selection life-threatening surgical complications can be considered rare.

In the absence of a generally accepted classification for outcome scores relating to surgical intervention for TMJ ankylosis in human or veterinary patients, a scoring system for TMJ ankylosis and pseudoankylosis surgical intervention success was proposed to better classify surgical successes and failures. We evaluated outcomes only in patients with long-term postoperative follow-up. The outcome score was excellent in 58.3% (7/13) of cases, good in 23.1% (3/13) of cases, and fair in 23.1% (3/13) of cases. Poor outcomes were not registered in this case series. The outcome score was not significantly associated with the diagnosis. While the possibility of complications must be considered and discussed with owners, these results support the use of current surgical techniques to improve the quality of life and function of animals affected by TMJ ankylosis and pseudoankylosis.

Restoration of ROM is one of the most critical aspects of determining surgical success. Within the current study, the mean (range) interincisal distance improved from 10.3 (2–30) to 65.3 (50–80) mm in dogs and from 5.71 (<1–20) to 33.6 (26–40) mm in cats. Of note, although improved, in most patients, the full expected ROM was not fully achieved. Interincisal opening in normal dogs and cats has been previously reported as 107 (range 40–180 mm) in dogs and 62 (range 41–84 mm) in cats (26). In previous studies, where animals had improved ROM but never regained normal ROM, quality of life, pain-free status, and functional mastication were still reported by all owners (23). Within the current study, 53.8% (7/13) of animals experienced Excellent response to treatment with 80% or greater return of ROM, and no animals experienced less than 20% return of ROM. Delayed return of ROM may be anticipated due to factors including inflammation of the TMJ and the surrounding bones, inflammation, and/ or contracture of the musculature, and potential nerve damage following surgical intervention. Appropriate time to evaluate treatment response should be permitted, and discussions are necessary to appropriately set owner expectations regarding supportive care, anticipated timeline for return to function, and incorporating measures to prevent contracture. Rehabilitation measures can be recommended to optimize recovery by restoring function, reducing pain, and preventing stiffness. These include anti-inflammatories, analgesics, and non-pharmacological therapies such as thermotherapy (cold and heat), electric stimulation, and physiotherapy (massage and stretching exercises), among other possibilities used in human patients (46). Pending patient temperament, stretching exercises similar to those performed in humans are possible with the adapted use of tools such as OraStretch (CranioMandibular Rehab, Wanaque, NJ). Yet, in most patients, focus on encouraging daily consistent maximum mouth opening through large food and toy play is often the most reliable and repeatable rehabilitation option.

Interestingly, the rate of complications and surgical outcomes was not associated with diagnosis. The authors expected to find fewer complications in cases of pseudoankylosis. Primary involvement of the TMJ in ankylosis is expected to require more extensive resection, with a suspected higher risk of iatrogenic damage and re-ankylosis. The lack of a significant association could be a result of the limited number of cases. However, other factors such as the surgical technique and planning can have a significant impact on complication rates and may have led to a confounding effect.

Furthermore, no association was found between 3D volume rendering and 3D printing in regards to the type of surgery performed, incidence of postoperative complications, postoperative malocclusions, the need for revision surgery, ROM improvement, or outcome score. CT or CBCT have been well established as the gold-standard imaging modalities for these disorders to provide proper diagnosis of the condition and allow for improved surgical planning (28, 29). Thus, this finding may be biased due to the small sample size, as 3D surgical planning has been shown to increase surgical predictability and safety in other surgical models, especially in animals of smaller size, such as the cat. It leads to improved surgical planning, more precise procedures, and reduced surgical time, which in combination can lead to lower complication rates and improved patient outcomes (14, 17, 18, 27, 30, 31).

Surgical technique was significantly associated with the occurrence of postoperative complications, the need for a revision surgery, and the outcome score. Given the heterogeneity of follow-up times, it is possible that complications were underestimated in the short and medium-term follow-up time groups. Regardless, even in short-term follow-up cases, complications such as acquired malocclusions and re-ankylosis were registered. EO was the most common surgical choice for pseudoankylosis cases and was associated with favorable outcomes, similar to a previous case report of EO in a cat (14). In contrast, SM was the technique associated with the highest number of complications, as they were noted to varying degrees in all five cases. SM also represented the procedure requiring the greatest frequency of revision surgeries. The complication rate, including the need for revision surgery, of SM was higher than that reported in a 2022 study, which included four cats (32). This may be a result of the limited number of cases in the 2022 report and the resultant limited representation of diagnosis and age of the animals. Cats receiving SM have been shown to experience complications of mandibular drift and malocclusion, but the current study elucidates the need to consider the likelihood of revision surgery when selecting SM compared to other surgical management options (32, 33). Regardless of procedure type, the frequency of malocclusion development following surgical correction highlights the importance of follow-up. Adequate clinical monitoring to identify the need for crown height reduction with endodontal therapy or selective dental extractions due to occlusal interference is an important component in achieving patient comfort and function. Clinical monitoring of ROM will also identify patients who would benefit from repeated diagnostic imaging to identify re-ankylosis. Within our case series, all patients developed re-ankylosis within 6 months of surgery. We recommended monthly evaluation of ROM for 6-month post-operatively and repeated diagnostic imaging if ROM decrease is noted.

While all cases requiring surgical revisions were mesocephalic animals less than 1 year of age, age was not significantly associated with the need for revision surgery. In young human and canine patients, mandibular regeneration following mandibulectomy has been documented and suspected to be due to regeneration via the periosteum (34, 35). Despite a lack of statistical significance between age and surgical revision in this study, the results of this case series may demonstrate that the creation of an insufficient gap defect during the procedure is not the primary cause of surgical failure, and that failure was ultimately a result of the regenerative abilities of the periosteum in young animals.

Compared to other procedures, GA was noted to result in the most varied outcome scores, which included Excellent, Good, and Fair results. Possible reasons for the variability include the extensive nature of the resection and minimal information regarding the recommended gap size in veterinary medicine. In humans, the recommended gap size for TMJ GA can range from 5 to 30 mm, depending on the severity of ankylosis, the use of interpositional materials, and the surgical technique used. Smaller gaps (5–9 mm) in humans are often sufficient when combined with interpositional materials and rigorous postoperative physiotherapy (36–39). One study in 16 adult dogs determined that the critical gap size in the mandible that would not spontaneously heal was 50 mm if the periosteum is preserved and 15 mm if the periosteum is removed (40); yet this gap size has not been corroborated with clinical patients with ankylosis that may have an accelerated healing potential, nor has a critical gap size ever been established in a juvenile population, which is the most likely group to undergo re-ankylosis post-surgery. Considered a salvage technique to restore adequate function, the need to completely excise the ankylosis site can naturally result in mandibular drift and malocclusion (17). The proximity of the TMJ to facial nerve branches also increases the risk of iatrogenic damage, especially in smaller-sized animals. When compared to the alternative, though, these are risks the operator and the client are often willing to accept to restore function to the patient, ideally with a single procedure.

The use of alloplastic materials instead of autologous tissues for IA has been utilized in human TMJ surgical interventions, with the goals of reducing donor site morbidity, decreasing ankylosis recurrence, and improving interincisal distance (22). Temporal myofascial flap utilization has been documented and shown to be successful in veterinary medicine as well, with temporal muscle atrophy being noted postoperatively in cats (23) and one dog (18). The requirement of GA and IA to resect the bony fusion in its entirety involves removal of a significant tissue mass, making these approaches more technically challenging and time-consuming than SM (32). In the initial surgical treatments that utilized IA, transposition of the temporal muscle or placement of fat was used as the interpositional material. IA resulted in improved outcome scores compared to GA, and no cases receiving IA required revision surgery. Due to the complexity and variability of the lesions, there is no uniform surgical treatment recommendation in humans for TMJ fusion; however, IA has been associated with a lower risk of re-ankylosis, improved mouth opening, and reduced pain compared to GA (21, 22, 41, 42). Alloplastic material development for use in veterinary medicine presents an opportunity for future research to improve surgical outcomes. IA cases resulted in excellent and good outcomes, but the majority were affected by muscle atrophy related to temporal muscle transposition. Development of alloplastic material options for veterinary patients could possibly result in outcomes with decreased recurrence of ankylosis as well as decreased patient morbidity. Additionally, a 2024 paper investigated TMJ replacement prosthesis in cadaveric cat and dog heads, displaying satisfactory function and mechanical stability (43). Research such as this can permit expansion of current human treatment modalities to veterinary patients, increasing surgical options and improving patient outcomes.

Review of the cases in this study confirms that positive outcomes are possible for both dogs and cats experiencing ankylosis or pseudoankylosis. Limitations of this study include the voluntary nature of case submission, the retrospective nature of the cases, lack of data on clinician decision making on surgical choice, varied clinician/skill levels in treatment, limited duration of postoperative follow-up information, and small sample size. Furthermore, the classification of complications as peri-operative, short-term, and long-term was challenging due to the range of follow-up time within the cohort as well as the variation within the literature as to what is classified as peri-operative, short-term, and long-term follow-up. Previous literature on oral complications defined short-term postoperative follow-up as either 25 h–30 days or 48 h–4 weeks, and long-term as >30 days (44, 45). As we had several patients who were lost to follow-up after 2 weeks, we broadly elected to refer to this group as short-term complications. We then added a medium-term complication group to capture patients who had more extended follow-up past the 2-week post-surgical recheck, but we did not feel they had long enough follow-up to identify re-ankylosis. Although the time to re-ankylosis is not documented in the literature, based on bone healing principles, we were concerned that 30 days would not be sufficient to capture this complication in all patients in a clinical setting. Thus, we elected to utilize 4 months as the inclusion criteria for identifying long-term complications, yet acknowledge that potentially a longer follow-up would have been more appropriate, and complications may have still been missed. We did not utilize a longer time period as we would have substantially decreased patients that met the inclusion criteria.

Despite these limitations, results of this study support that SM has the highest rate of postoperative complications and should be reserved only for cases at very high risk of iatrogenic complications with more extensive procedures (such as entering the skull base or very high risk of hemorrhage with no ability to remedy at the facility). When deciding upon the use of interpositional materials, the potential complication of atrophy related to temporal muscle transposition may be a worthwhile compromise to improve overall chances of good or excellent outcomes, and the use of patient fat and/or the development of alloplastic interpositional materials is a worthwhile endeavor for future research.

## Data Availability

The raw data supporting the conclusions of this article will be made available by the authors, without undue reservation.

## References

[1] Arzi B Cissell DD Verstraete FJ Kass PH DuRaine GD Athanasiou KA. Computed tomographic findings in dogs and cats with temporomandibular joint disorders: 58 cases (2006-2011). J Am Vet Med Assoc. (2013) 242:69–75. doi: 10.2460/javma.242.1.69 23234284 PMC3747040

[2] Nickel R Schummer A Seiferle E In: Nickel R Schummer A Seiferle E, editors. The anatomy of the domestic animals, vol. 1. 5th ed. Berlin: Verlag Paul Parey Press (1986). 172–3.

[3] Evans HE de Lahunta A. Ligaments and joints of the skull. In: Miller’s anatomy of the dog, Evans H. E. Lahunta A. de, Eds., Elsevier Saunders, St. Louis (2013). 161–162.

[4] Neville BW Damn DD Allen CM Bouquot JE. Facial pain and neuromuscular diseases In: Neville BW Damm DD Chi AC, editors. Oral and maxillofacial pathology. 2nd ed. Philadelphia: W.B. Saunders (2002). 741–59.

[5] Petrowski CG. Bone ankylosis In: Tamimi D Hatcher DC, editors. Specialty imaging temporomandibular joint. 1st ed. Philadelphia: Elsevier (2016). 524–5.

[6] Hatcher DC Koenig LJ. Fibrous ankylosis In: Tamimi D Hatcher DC, editors. Specialty imaging temporomandibular joint. 1st ed. Philadelphia: Elsevier (2016). 522–3.

[7] Bennett D Campbell JR. Mechanical interference with lower jaw movement as a complication of skull fractures. J Small Anim Pract. (1976) 17:747–51. doi: 10.1111/j.1748-5827.1976.tb06938.x, PMID: 1011800

[8] van Ee RT Pechman RD. False ankylosis of the temporomandibular joint in a cat. J Am Vet Med Assoc. (1987) 191:979–80. doi: 10.2460/javma.1987.191.08.979, PMID: 3679994

[9] Sullivan M. Temporomandibular ankylosis in the cat. J Small Anim Pract. (1989) 30:401–5 14. doi: 10.1111/j.1748-5827.1989.tb01589.x

[10] Anderson A. What is your diagnosis? Ankylosis of the left temporomandibular joint. J Small Anim Pract. (1997) 38:279–95. 9239627

[11] Rickert S Kass P Verstraete F. Temporomandibular joint pathology of wild carnivores in the western USA. Front Vet Sci. (2021) 8:657381. doi: 10.3389/fvets.2021.657381, PMID: 33898548 PMC8063859

[12] Meomartino L Fatone G Brunetti A Lamagna F Potena A. Temporomandibular ankylosis in the cat: a review of seven cases. J Small Anim Pract. (1999) 40:7–10. doi: 10.1111/j.1748-5827.1999.tb03245.x, PMID: 10092035

[13] Erol B Tanrikulu R Görgün B. A clinical study on ankylosis of the temporomandibular joint. J Craniomaxillofac Surg. (2006) 34:100–6. doi: 10.1016/j.jcms.2005.07.008, PMID: 16423530

[14] Larguier L Jamet N. False ankylosis of the temporomandibular joint in a cat. Correction by partial zygomatic arch resection. Vet Comp Orthop Traumatol. (2015) 28:455–8. doi: 10.3415/VCOT-15-01-0010, PMID: 26383183

[15] Roychoudhury A Parkash H Trikha DA. Functional restoration by gap arthroplasty in temporomandibular joint ankylosis: a report of 50 cases. Oral Surg Oral Med Oral Pathol Oral Radiol Endod. (1999) 87:166–9. doi: 10.1016/s1079-2104(99)70267-2, PMID: 10052370

[16] Manganello-Souza LC Mariani PB. Temporomandibular joint ankylosis: report of 14 cases. Int J Oral Maxillofac Surg. (2003) 32:24–9. doi: 10.1054/ijom.2002.0308, PMID: 12653228

[17] Aghashani A Verstraete FJM Arzi B. Temporomandibular joint gap arthroplasty in cats. Front Vet Sci. (2020) 7:482. doi: 10.3389/fvets.2020.00482, PMID: 32903530 PMC7438723

[18] Renner E Thatcher G. Combined gap and Interpositional arthroplasty utilizing three-dimensional printed model in a dog with temporomandibular joint Ankylosis and Pseudoankylosis. J Vet Dent. (2022) 39:284–9. doi: 10.1177/08987564221100670, PMID: 35642268

[19] Thangavelu A Santhosh Kumar K Vaidhyanathan A Balaji M Narendar R. Versatility of full thickness skin-subcutaneous fat grafts as interpositional material in the management of temporomandibular joint ankylosis. Int J Oral Maxillofac Surg. (2011) 40:50–6. doi: 10.1016/j.ijom.2010.06.025, PMID: 20952163

[20] Su-Gwan K. Treatment of temporomandibular joint ankylosis with temporalis muscle and fascia flap. Int J Oral Maxillofac Surg. (2001) 30:189–93. doi: 10.1054/ijom.2001.0047, PMID: 11420899

[21] Bansal S Verma DK Rai M Sorake A Kaur C. Gap arthroplasty or Interpositional arthroplasty for the management of TMJ Ankylosis? A prospective randomized comparative multicenter clinical trial. J Maxillofac Oral Surg. (2019) 18:567–71. doi: 10.1007/s12663-018-1150-z, PMID: 31624438 PMC6795650

[22] Desai H Pande N Jawdekar A. Comparison of surgical outcomes related to interpositional arthroplasty materials used in patients with temporomandibular joint ankylosis: a systematic review and meta-analysis. Br J Oral Maxillofac Surg. (2022) 60:1023–34. doi: 10.1016/j.bjoms.2022.05.005, PMID: 35906111

[23] Mestrinho K Gawor J Serrano A Niza M. Superficial temporal myofascial flap application in temporomandibular joint arthroplasty in a cat. J Feline Med Surg Open Rep. (2015) 1:2055116915593965. doi: 10.1177/2055116915593965 PMC5362026 28491369

[24] American Veterinary Dental College Nomenclature. Occlusal abnormalities (2025). Available online at: https://avdc.org/avdc-nomenclature (Accessed March 17, 2025).

[25] Strøm P Arzi B Cissell D Verstraete FJM. Ankylosis and pseudoankylosis of the temporomandibular joint in 10 dogs (1993-2015). Vet Comp Orthop Traumatol. (2016) 29:409–15. doi: 10.3415/VCOT-15-11-0189, PMID: 27439984

[26] Gracis M Zini E. Vertical mandibular range of motion in anesthetized dogs and cats. Frontiers Vet Sci. (2016) 3:51. doi: 10.3389/fvets.2016.00051, PMID: 27446939 PMC4923261

[27] Winer JN Verstraete FJM Cissell DD Lucero S Athanasiou KA Arzi B. The application of 3-dimensional printing for preoperative planning in oral and maxillofacial surgery in dogs and cats. Vet Surg. (2017) 46:942–51. doi: 10.1111/vsu.12683, PMID: 28688157

[28] Bar-Am Y Pollard RE Kass PH Verstraete FJM. The diagnostic yield of conventional radiographs and computed tomography in dogs and cats with maxillofacial trauma. Vet Surg. (2008) 37:294–9. doi: 10.1111/j.1532-950X.2008.00380.x, PMID: 18394078

[29] Cissell DD Hatcher D Arzi B Verstraete FJM. Diagnostic imaging in oral and maxillofacial surgery In: Verstraete FJM Lommer MJ Arzi B, editors. Oral and maxillofacial surgery in dogs and cats. St. Louis: Elsevier (2020). 56–64.

[30] Huang YH Lee B Chuy JA Goldschmidt SL. 3D printing for surgical planning of canine oral and maxillofacial surgeries. 3D Print Med. (2022) 8:17. doi: 10.1186/s41205-022-00142-y, PMID: 35678954 PMC9178851

[31] Thatcher GP Soukup JW. Virtual surgical planning and 3D printing in veterinary dentistry and oromaxillofacial surgery. Vet Clin North Am Small Anim Pract. (2022) 52:221–34. doi: 10.1016/j.cvsm.2021.09.009, PMID: 34838251

[32] Villamizar-Martinez L Ferro1 D Carvalho V Ferreira J Reiter A. Caudal and middle segmental mandibulectomies for the treatment of unilateral temporomandibular joint ankylosis in cats. J Feline Med Surg Open Rep. (2022) 8:20551169221086438. doi: 10.1177/20551169221086438 PMC8978324 35386208

[33] Villamizar-Martinez LA Chia H Robertson JB Villegas CM Reiter AM. Comparison of unilateral rostral, middle and caudal segmental mandibulectomies as an alternative treatment for unilateral temporomandibular joint ankylosis in cats: an ex vivo study. J Feline Med Surg. (2021) 23:783–93. doi: 10.1177/1098612X20977134, PMID: 33289444 PMC10812199

[34] Budal J. The osteogenic capacity of the periosteum. Oral Surg Oral Med Oral Pathol. (1979) 47:227–9. doi: 10.1016/0030-4220(79)90145-2, PMID: 283353

[35] Wright A Peralta S Fiani N. Case report: spontaneous mandibular body regeneration following unilateral subtotal mandibulectomy in a 3-month-old French bulldog. Front Vet Sci. (2023) 10:1281232. doi: 10.3389/fvets.2023.1281232 37901099 PMC10600472

[36] Babu L Jain MK Ramesh C Vinayaka N. Is aggressive gap arthroplasty essential in the management of temporomandibular joint ankylosis? A prospective clinical study of 15 cases. Br J Oral Maxillofac Surg. (2103) 51:473–8. doi: 10.1016/j.bjoms.2012.11.004 23219020

[37] Ma J Liang L Jiang H Gu B. Gap arthroplasty versus interpositional arthroplasty for temporomandibular joint ankylosis: a meta-analysis. PLoS One. (2015) 10:e0127652. doi: 10.1371/journal.pone.0127652, PMID: 26010224 PMC4444315

[38] Otake Y Nogami S Chiba M Odashima K Takeda Y Miyashia H. Low level gap arthroplasty using ultrasonic bone cutting device for recurrent temporomandibular joint ankylosis—case report. J Oral Maxillofac Surg Med Pathol. (2022) 34:749–53. doi: 10.1016/j.ajoms.2022.04.002

[39] Temerek AT. Conservative gap arthroplasty in temporomandibular ankylosis not involving the sigmoid notch: a selected age group study. Br J Oral Maxillofac Surg. (2016) 54:38–43. doi: 10.1016/j.bjoms.2016.02.030 26972420

[40] Huh JY Choi BH Kim BY Lee SH Zhu SJ Jung JH. Critical size defect in the canine mandible. Oral Surg Oral Med Oral Pathol Oral Radiol Endod. (2005) 100:296–301. doi: 10.1016/j.tripleo.2004.12.015, PMID: 16122656

[41] He CH. Management of traumatic temporomandibular joint ankylosis: a case report. Oral Maxillofac Surg Cases. (2024) 10:100344. doi: 10.1016/j.omsc.2023.100344

[42] Dimitroulis G. The interpositional dermis-fat graft in the management of temporomandibular joint ankylosis. Int J Oral Maxillofac Surg. (2004) 33:755–60. doi: 10.1016/j.ijom.2004.01.012, PMID: 15556322

[43] Arzi B Weed M Garcia TC Goldschmidt SL Marcellin-Little DJ. Kinematic performance of a novel temporomandibular joint replacement prosthesis under bite-force conditions in dogs and cats. Am J Vet Res. (2024) 85:ajvr.24.01.0009. doi: 10.2460/ajvr.24.01.0009, PMID: 38640955

[44] MacLellan RH Rawlinson JE Rao S Worley DR. Intraoperative and postoperative complications of partial maxillectomy for the treatment of oral tumors in dogs. J Am Vet Med Assoc. (2018) 252:1538–47. doi: 10.2460/javma.252.12.1538, PMID: 29889637

[45] Rigby BE Malott K Sample SJ Hetzel SJ Scott J Soukup JW. Impact of the surgical environment on the incidence, timing, and severity of complications associated with oromaxillofacial oncologic surgery in dogs. Front Vet Sci. (2021). doi: 10.3389/fvets.2021.760642 PMC8718541 34977206

[46] De Meurechy NKG Loos PJ Mommaerts MY. Postoperative physiotherapy after open temporomandibular joint surgery: a 3-step program. J Oral Maxillofac Surg. (2019) 77:932–50. doi: 10.1016/j.joms.2018.12.027, PMID: 30689965

